# Sex differences in brain glucose metabolism in alzheimer’s disease: A voxel-based study

**DOI:** 10.1007/s11357-025-01872-7

**Published:** 2025-09-05

**Authors:** Matilde Nerattini, Elisabetta Maria Abenavoli, Francesca Caramelli, Giulia Giacomucci, Benedetta Nacmias, Enrico Mossello, Valentina Bessi, Valentina Berti

**Affiliations:** 1 Department of Experimental and Clinical Biomedical Sciences, Nuclear Medicine Unit, University of Florence, Florence, Italy; 2 Careggi University Hospital, Nuclear Medicine Unit, Florence, Italy; 3 Geriatric Unit, Ospedale San Jacopo Pistoia, Pistoia, Italy; 4 Department of Experimental and Clinical Medicine, Research Unit of Medicine of Ageing, University of Florence, Florence, Italy; 5 Department of Neuroscience, Psychology, Drug Research and Child Health, University of Florence, Psychology, Italy; 6 IRCCS Fondazione Don Carlo Gnocchi, Florence, Italy; 7 SOD Neurologia, Azienda Ospedaliero Universitaria Careggi, Florence, Italy

**Keywords:** Alzheimer’s disease, Positron emission tomography, FDG, Sex, Women

## Abstract

A growing body of evidence shows significant sex differences in Alzheimer’s Disease (AD) epidemiology, clinical presentation, and pathology burden; however, sex differences in neuroimaging biomarkers remain underexplored, prompting recent calls to action for more targeted research in this field. We analyzed static brain positron emission tomography (PET) imaging with 2-[^18^F] fluoro-2-deoxy-D-glucose (FDG) from 247 elderly individuals with AD dementia, including 151 women and 96 men. Voxel-based analysis was used to detect reductions in FDG uptake in each sex relative to a publicly shared normative database and to identify sex differences in FDG uptake within the AD cohort. Both sexes exhibited glucose hypometabolism in AD-vulnerable regions, including the parieto-temporal cortex, posterior cingulate, hippocampus, parahippocampal gyrus, and frontal lobes (*P*_*FWE*_ ≤ 0.001 in women and ≤ 0.013 in men). Sex differences in regional FDG uptake were observed in both directions, with greater hypometabolism in limbic and frontal regions in women (*P*_*FWE*_ ≤ 0.023) and in parietal cortices in men (*P*_*FWE*_ ≤ 0.008). The sex-specific distribution of hypometabolism, with more pronounced anterior involvement in women and posterior involvement in men, aligns with known differences in brain reserve and hormone-sensitive regions. This pattern suggests that neurophysiological and neuroendocrine aging may contribute to AD neuropathology in a sex-dependent manner. Recognizing these variations could refine diagnostic approaches and inform the development of sex-specific therapeutic strategies.

## Introduction

Alzheimer’s Disease (AD) is the most common form of dementia, affecting over 24 million people worldwide [1]. A growing body of evidence highlights significant sex differences in AD epidemiology, clinical presentation, and pathology burden [2–4]. Consequently, early intervention strategies that incorporate sex-specific considerations have been proposed as a promising approach for early diagnosis and to mitigate the AD epidemic.

Women account for approximately two-thirds of AD cases [5], a disparity that may not be fully explained by greater female longevity [1]. Several mechanisms have been proposed to explain this sex difference, including risk factors that are (i) equally prevalent in both sexes but exert a greater effect in women, (ii) more common in women than men, and (iii) exclusive to women [6]. Particularly, the apolipoprotein E (APOE-ε4) allele, the strongest genetic risk factor for late-onset AD, has been shown to confer a greater risk in women, leading to earlier disease onset and higher pathology burden [7, 8]. Additionally, differences in cognitive reserve—partially influenced by education and occupational attainment, which have historically been lower in women—may contribute to this increased risk [9]. Hormonal factors also play a crucial role, as menopause-related estrogen decline has been implicated in increased AD susceptibility among women [10, 11].

Pathological and neuroimaging studies suggest that although men tend to exhibit more severe AD-related brain changes [12, 13], women are more likely to develop clinical dementia at comparable pathology levels [14]. Barnes et al. found that for every unit increase in AD pathology, women were significantly more likely than men to exhibit clinical dementia [14]. Similarly, Rowe et al. used amyloid positron emission tomography (PET) with Pittsburgh compound B to demonstrate that, despite comparable cognitive impairment, men exhibited a higher amyloid load than women [12].

2-[^18^F] fluoro-2-deoxy-D-glucose (FDG) PET studies have demonstrated high accuracy in identifying AD dementia, marking a milestone in clinical practice [15]. However, sex differences have not been formally integrated into diagnostic criteria. Using FDG PET, sex-related differences in brain glucose metabolism have been explored during healthy aging [16–20], with some evidence suggesting greater age-related reductions in frontal metabolism in men compared to women [21], although findings remain inconsistent [16–20]. In AD patients, research has primarily focused on the interaction between sex, glucose metabolism, and cognitive reserve [13, 21]. A voxel-based analysis suggested that men with AD exhibit greater reductions in glucose metabolism than women at comparable clinical severity, potentially reflecting greater brain reserve in men [13]. Additionally, sex differences were found in the effects of education and occupation on regional FDG PET metabolic activity and functional connectivity, with opposing directions during healthy aging and AD [21]. Topographically, cognitive reserve was found to modulate glucose metabolism in the anterior executive and socio-affective networks in women, and in the posterior associative regions in men [21].

Despite these emerging insights, the metabolic patterns underlying sex differences in AD remain incompletely understood. Addressing these gaps is crucial for optimizing early diagnosis and developing personalized therapeutic strategies.

Therefore, the aim of the present study is to elucidate sex differences in AD-related metabolic patterns using static FDG PET imaging. By examining regional variations in FDG uptake, this study seeks to improve our understanding of sex-specific disease trajectories and their implications for clinical management.

## Methods

### Participants

This is a retrospective, single-center observational study of elderly men and women (Table 1), with late-onset Alzheimer’s disease (AD). The study population was drawn from individuals who underwent brain FDG PET at the Nuclear Medicine Unit of the Azienda Ospedaliero-Universitaria Careggi in Florence, Italy, between 2014 and 2022. Only participants with a confirmed clinical diagnosis of Alzheimer’s disease within approximately two years of FDG PET imaging were included in the analysis, and individuals meeting clinical criteria for other neurological or psychiatric conditions were excluded. Specifically, inclusion criteria were: (i) a clinical diagnosis of AD dementia according to the National Institute on Aging (NIA) criteria [22], or (ii) biomarker-confirmed AD based on the Revised Criteria of the Alzheimer’s Association Workgroup [22]. Biomarker confirmation, when available, included amyloid PET and/or cerebrospinal fluid (CSF) analysis (Aβ1–42, Aβ1–42/1–40 ratio, t-tau, p-tau). Details on biomarker acquisition are described elsewhere [23–25]. The presence of depression was assessed based on self-reported history of antidepressant use (ever vs. never).

**Table 1:** Patients’ characteristics

	Men	Women	Effect size^1^	*p-*value
N	96	151		
Age, years	70.97 ± 8.22	69.42 ± 8.73	0.18	0.160
MMSE	23.21 ± 5.52	23.11 ± 4.81	0.02	0.884
Education, years	11.68 ± 4.41	10.30 ± 4.59	0.31	**0.019**
Depression, % positive	37.5%	68.8%	0.311	**0.020**

Values are mean (standard deviations) unless otherwise specified

^1^Effect sizes are Cohen’s d for continuous variables and Cramere’s V for categorical variables

Significant results are shown in bold

### Brain imaging and analyses

Static PET scans were acquired 30–40 min after ^18^F-FDG administration (3.7 MBq/kg) according to EANM guidelines for brain imaging [26]. Images were obtained on a PET/CT scanner (Philips Gemini TF 16 PET/CT) and reconstructions were performed using 3D LOR iterative algorithm reconstruction (FOV: 256, matrix: 128 × 128, voxel dimensions: 2 × 2 × 2 mm). CT acquisition for attenuation correction was performed on spiral 16 slices CT with a slice thickness of 2 mm.

FDG PET data were analyzed using a semiquantitative approach with statistical parametric mapping (SPM12) [27] on MATLAB (MathWorks Inc., Sherborn, MA, USA), following standardized protocols [27–29]. SUV images, computed as the ratio between tissue activity concentration (kBq/mL) and the injected dose (kBq) normalized by body weight (g) were used as input for analysis. Then scans were anonymized, manually reoriented, setting the origin to the anterior commissure, normalized according to the dementia-specific ^18^F-FDG PET template [28], and then smoothed using an isotropic Gaussian kernel of 8 mm full-width at half-maximum (FWHM). For statistical analysis, proportional scaling to the global mean was applied during model estimation to account for potential factors of interindividual variability [28, 29].

Comparisons were performed using an independent-samples ANOVA design: (i) between AD patient groups and normative controls provided by the Italian Association of Nuclear Medicine (AIMN) [30]; and (ii) between AD patients, with age and Mini Mental State Examination (MMSE) as nuisance variables and activity proportionally scaled to the global mean value. The significance threshold was set at *p* < *0.05*, Family Wise Error (FWE) corrected for multiple comparisons. Only clusters containing more than 16 voxels were deemed to be significant (e.g., twice the FWHM) to further reduce the likelihood of Type I errors [31]. Anatomical location of regions reaching significance was described using MNI coordinates and Automated Anatomical Labelling atlas 3 (AAL3) regions [32].

### Statistical analyses

All statistical analyses have been performed with software IBM® SPSS® Statistics. Comparisons between variables have been performed using chi-square test or Welch’s t-test, as appropriate. Statistical significance was set at *p* < *0.05*.

## Results

A total of 247 participants met our inclusion criteria and were available for analysis, including 151 women and 96 men. Participants’ characteristics are given in Table 1. There were no sex differences for age and cognitive measures, i.e. MMSE. The female group included a significantly higher percentage of patients with depression and significantly lower education scores than the male group (p < 0.05).

### Comparisons between AD patients and healthy controls

**Fig. 1:**
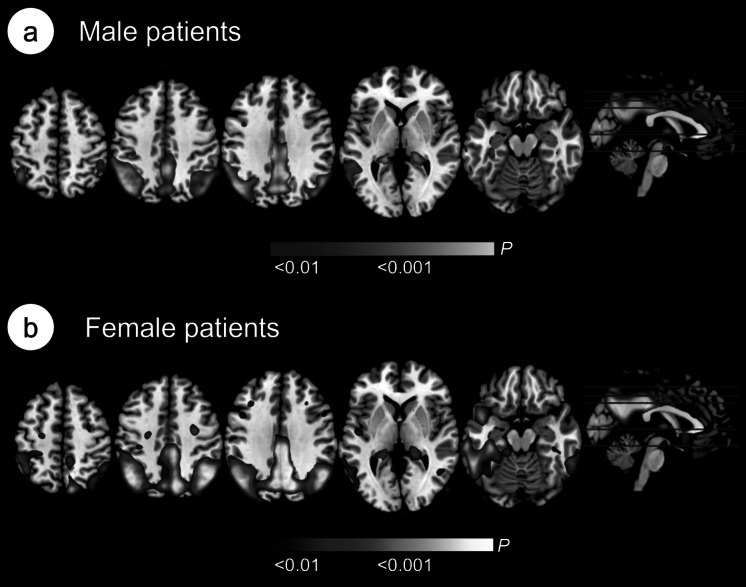
Hypometabolic patterns in male and female AD patients versus healthy controls. Statistical parametric T maps (SPMs) of significant glucose hypometabolism in male AD patients (in blue, a) and female AD patients (in purple, b) as compared to healthy controls. SPMs are generated at P < 0.05 corrected for Family-Wise Type Error (FWE) and displayed onto a standardized MRI using color-coded scales with corresponding T values

Male AD patients showed lower FDG uptake compared to healthy controls in inferior parietal lobule, parahippocampal gyrus, and angular gyrus, bilaterally; in middle temporal gyrus, middle cingulate cortex, precuneus, and superior, middle and inferior frontal cortices of the left hemisphere; and in posterior cingulate cortex and hippocampus of the right hemisphere (*P*_*FWE*_ ≤ 0.013, Table 2 and Fig. 1a).

**Table 2:** Clusters of significant glucose hypometabolism in male AD patients as compared to controls

Cluster extent (voxels)	Anatomical region (AAL)	P_FWE_*	T	MNI coordinates x, y, z (mm)
12170	Inferior parietal lobule, left	< 0.001	11.49	−52 −60 38
	Middle temporal gyrus, left	< 0.001	11.31	−62 −48 −8
	Angular gyrus, left	< 0.001	10.29	−58 −58 18
5836	Middle cingulate, left	< 0.001	11.17	−4 −26 30
	Precuneus, left	< 0.001	11.09	0 −48 36
	Posterior cingulate, right	< 0.001	10.20	8 −56 30
4064	Angular gyrus, right	< 0.001	8.09	52 −62 44
	Angular gyrus, right	< 0.001	7.84	52 −66 32
	Inferior parietal lobule, right	< 0.001	7.13	38 −56 44
458	Superior frontal gyrus, left	< 0.001	6.76	−26 8 68
	Middle frontal gyrus, left	< 0.001	5.66	−44 14 44
104	Parahippocampal gyrus, left	< 0.001	6.47	−20 4 −30
27	Hippocampus, right	0.001	5.55	14 −2 −16
	Parahippocampal gyrus, right	0.013	4.89	22 6 −26
121	Inferior frontal gyrus, left	0.001	5.54	−52 24 24
	Inferior frontal operculum, left	0.004	5.19	−56 16 16

^*^P < *0*.*05* corrected for Family-Type Wise Error (FWE). Analyses were adjusted by age, MMSE, and global mean FDG uptake

*AAL *Automated Anatomical Labeling*; MNI *Montreal Neurological Institute

Female AD patients showed lower FDG uptake compared to healthy controls in anterior cingulate cortex, bilaterally; precuneus, hippocampus, parahippocampal gyrus, inferior parietal lobule, angular gyrus, and inferior temporal gyrus of the right hemisphere; and posterior cingulate cortex, superior, middle and orbital-frontal cortices, and superior temporal gyrus of the left hemisphere (*P*_*FWE*_ ≤ 0.001, Table 3, Fig. 1b).

**Table 3:** Clusters of significant glucose hypometabolism in female AD patients as compared to controls

Cluster extent (voxels)	Anatomical region (AAL)	P_FWE_*	T	MNI coordinates x, y, z (mm)
13348	Precuneus, right	< 0.001	11.30	8 −56 30
	Posterior cingulate, left	< 0.001	11.27	0 −52 30
	Posterior cingulate, left	< 0.001	11.21	0 −32 32
594	Hippocampus, right	< 0.001	8.30	18 −36 −2
	Parahippocampal gyrus, right	< 0.001	7.38	30 −40 −4
1258	Inferior parietal, lobule, right	< 0.001	6.79	52 −66 36
	Angular gyrus, right	< 0.001	6.79	52 −62 44
187	Anterior cingulate, left	< 0.001	6.51	−2 16 24
	Anterior cingulate, right	< 0.001	5.91	2 8 28
260	Inferior temporal gyrus, right	< 0.001	6.07	62 −46 −20
43	Parahippocampal gyrus, right	< 0.001	5.96	24 4 −28
34	Superior frontal gyrus, left	< 0.001	5.95	−24 −14 48
158	Middle frontal gyrus, left	< 0.001	5.84	−46 12 40
94	Superior temporal gyrus, left	< 0.001	5.65	−44 −6 −8
27	Orbitofrontal gyrus, left	0.001	5.59	−32 58 −14
52	Inferior temporal gyrus, right	0.001	5.57	46 −32 −20

^*^P < *0*.*05* corrected for Family-Type Wise Error (FWE). Analyses were adjusted by age, MMSE, and global mean FDG uptake

*AAL *Automated Anatomical Labeling*; MNI *Montreal Neurological Institute

### Comparison between AD patients

**Fig. 2:**
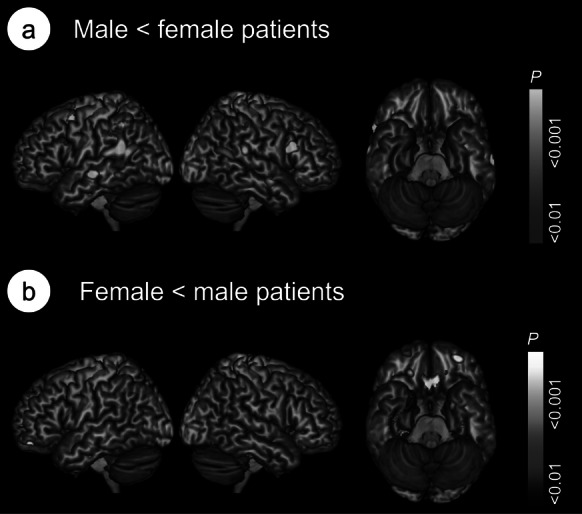
Sex differences in glucose metabolism in the AD population. Statistical parametric T maps (SPMs) of significant glucose hypometabolism in male AD patients compared to female AD patients (in blue, a); and in female AD patients compared to male AD patients (in purple, b). SPMs are generated at P < 0.05 corrected for Family-Wise Type Error (FWE) and displayed onto a standardized 3D volume-rendered MRI using color-coded scales with corresponding T values

Male AD patients exhibited lower FDG uptake compared to female AD patients in supramarginal gyrus and precuneus of the left hemisphere (*P*_*FWE*_ ≤ 0.008, Table 4, Fig. 2a).

**Table 4:** Clusters of significant glucose hypometabolism in male AD patients as compared to female AD patients

Cluster extent (voxels)	Anatomical region (AAL)	P_FWE_*	T	MNI coordinates x, y, z (mm)
68	Supramarginal gyrus, left	0.002	5.37	−66 −40 42
33	Precuneus, left	0.008	5.02	2 −36 78

^*^P < *0*.*05* corrected for Family-Type Wise Error (FWE). Analyses were adjusted by age, MMSE, and global mean FDG uptake

*AAL *Automated Anatomical Labeling;* MNI *Montreal Neurological Institute

Female AD patients exhibited lower FDG uptake compared to male patients in parahippocampal gyrus and inferior temporal gyrus, bilaterally; hippocampus and orbitofrontal cortex of the right hemisphere; and anterior cingulate cortex of the left hemisphere (*P*_*FWE*_ ≤ 0.023, Table 5, Fig. 2b).

**Table 5:** Clusters of significant glucose hypometabolism in female AD patients as compared to male AD patients

Cluster extent (voxels)	Anatomical region (AAL)	P_FWE_*	T	MNI coordinates x, y, z (mm)
205	Hippocampus, right	< 0.001	5.73	34 −48 −32
	Parahippocampal gyrus, right	0.023	4.76	22 −62 −28
84	Inferior temporal gyrus, right	< 0.001	5.70	50 −18 −42
	Fusiform gyrus, right	0.009	4.98	40 −10 −48
121	Parahippocampal gyrus, left	0.004	5.17	−26 −46 −32
	Inferior temporal gyrus, left	0.016	4.85	−22 −58 −30
79	Anterior cingulate, left	0.004	5.15	−6 32 −10
27	Medial orbitofrontal gyrus, right	0.009	4.98	18 32 −18

^*^P < *0*.*05* corrected for Family-Type Wise Error (FWE). Analyses were adjusted by age, MMSE, and global mean FDG uptake

*AAL *Automated Anatomical Labeling*; MNI *Montreal Neurological Institute

## Discussion

This voxel-based study identifies regional sex differences in glucose metabolism on FDG PET in elderly individuals with AD dementia. Both sexes exhibited reduced FDG uptake compared to controls in parieto-temporal, limbic, and frontal regions, consistent with the established AD hypometabolic pattern [15]. Notably, we observed a sex-specific regional distribution of hypometabolism: in women, reductions were more pronounced in anterior regions, while in men, they were more prominent in posterior areas, suggesting distinct regional vulnerabilities to AD pathology. These findings were independent of age and MMSE, and adjusted for global mean FDG PET uptake.

Our results expand on exiguous existing literature by identifying sex differences in AD-related hypometabolism. In men, our findings align with previous evidence of lower glucose metabolism in male vs. female AD patients on FDG PET [13]. Additionally, the observed male-specific hypometabolism in the supramarginal gyrus and precuneus is consistent with studies reporting greater male vulnerability to AD-related changes in parietal regions, as evidenced by regional smaller gray matter volumes on MRI [33] and lower cerebral blood flow (CBF) on SPECT [34] in men vs. women with AD.

Interestingly, findings of lower glucose metabolism in female AD patients compared to men are novel. Differences from a previous FDG PET study showing no areas of significant female-specific hypometabolism in AD patients may stem from our larger sample size (approximately three times that of the prior study) [13]. Alternatively, our female patients may have been at a more advanced disease stage, as suggested by hypometabolism in frontal cortices relative to healthy controls—regions typically affected later in AD progression [35]. This pattern is consistent with studies reporting worse imaging outcomes in female vs. male AD patients, as shown using amyloid- and tau-PET [36], which detect pathology earlier than neurodegeneration markers [1]. Additionally, our findings of female-specific limbic and frontal hypometabolism align with SPECT studies showing lower CBF in these regions in female vs. male AD patients [34].

Altogether, our findings of frontal and limbic hypometabolism in women and parietal hypometabolism in men may reflect previously reported sex differences in the spatial distribution of brain reserve [21]. A prior FDG PET study demonstrated that education and occupation—key determinants of cognitive reserve—modulate brain glucose metabolism in both healthy controls and AD patients in a sex-specific manner [21]. Specifically, cognitive reserve correlated with glucose metabolism in frontal executive and limbic socio-affective networks in women and with parietal associative cortices and dorsal default mode network in men [21]. These correlations were positive in healthy controls but reversed in AD [21], suggesting that regions with higher baseline reserve may experience greater metabolic decline in AD. The overlap between our hypometabolism findings in AD and these sex-specific reserve networks supports this hypothesis, warranting further investigation into the relationship between brain reserve and AD vulnerability.

A potential mechanism underlying the observed sex differences could be the modulation of glucose metabolism by sex hormones. Higher levels of estrogens in women and testosterone in men have been shown to support brain health [11, 37–40], while lower levels increase AD risk [41–44]. Preliminary evidence suggests similar roles for gonadotropins in both sexes [45–48]. Regionally, testosterone effects have been reported, not exclusively, in parietal regions in men, while estrogen primarily influences frontal and limbic areas in women, as evidenced by hormone-receptor expression [49], correlations with imaging regional outcomes [10, 40, 50], and more indirect cognitive associations (e.g., visuospatial performance in men [51], memory performance in women [52]). Our results align with these findings and further support the role of sex hormones in shaping sex-specific AD-related brain changes.

Furthermore, menopause plays a critical role in female AD risk and pathophysiology [37]. Postmenopausal women exhibited lower glucose metabolism, greater amyloid burden, and reduced gray and white matter volumes compared to both premenopausal women and age-matched men [53]. Recent in vivo evidence suggested that estrogen receptor density increases across the menopause transition, likely as a compensatory response to ovarian estrogen loss [54]. Given that these effects were also observed in hippocampal and frontal regions [53, 54]—areas implicated in our study—and that our female AD patients are likely postmenopausal due to their age, our findings support the increased vulnerability of the hippocampus and frontal cortices to AD changes induced by the menopause transition, thereby possibly strengthening the understanding of menopause’s impact on female AD pathophysiology.

Finally, women showed a higher prevalence of depression and lower education levels than men, consistent with prior epidemiological data [55]. As both are recognized AD risk factors, their higher prevalence in women may partly contribute to sex-related differences in AD vulnerability. Further studies are warranted to clarify their role in shaping sex-specific disease expression.

### Strengths and limitations

This study has several strengths. First, we used state-of-the-art voxel-based analysis in a relatively large sample, which enabled us to detect subtle yet significant sex differences in AD metabolism. While age-related frontal hypometabolism is well-documented in the literature [16–19], we controlled for age effects in all analyses. Notably, researchers suggested that age-related frontal hypometabolism is more pronounced in men [21], whereas we found greater frontal metabolism reductions in women in AD, further supporting the specificity of our findings. Even if residual age effects were present, they would likely lead to underestimation rather than overestimation of female-specific hypometabolism.

Second, our study provides clinically relevant insights by investigating sex differences in brain FDG uptake in real-world AD patients. Importantly, the female-specific frontal hypometabolism observed falls outside the classical AD pattern [15], raising questions about its potential diagnostic significance in women.

However, several limitations should be acknowledged. First, we did not stratify patients by AD variant, preventing evaluation of sex differences within specific AD subtypes. Second, APOE status was unavailable, despite its documented sex-dependent effects on AD risk [7]. Future studies should explore interactions between APOE-ε4, sex, and glucose metabolism in AD.

Moreover, FDG-PET was not systematically performed in all patients with suspected Alzheimer’s disease but was instead ordered in selected cases with diagnostic uncertainty, in line with international guidelines [15]. While this reflects routine clinical practice, it may introduce selection bias and limit generalizability. Replication in systematically imaged research cohorts is recommended.

The use of static FDG PET scans and semiquantitative voxel-based methods, although clinically relevant and formally recommended for the assessment of neurodegenerative disorders, lacks the quantitative precision of full kinetic modeling. Future research should incorporate dynamic imaging protocols to improve accuracy and sensitivity.

Additionally, we lacked full clinical and neuropsychological assessments and a biological diagnosis of all participants. However, no sex differences in MMSE scores were observed, suggesting comparable clinical severity between groups. Future research incorporating detailed neuropsychological evaluations and comprehensive pathological biomarker assessments is warranted to confirm these findings.

Moreover, the cross-sectional design of the study limits causal interpretation. Specifically, we were unable to determine whether depressive symptoms represent a risk factor for, or a consequence of, AD-related pathology. Longitudinal research is needed to establish temporal directionality.

Finally, sex hormone levels were not available, preventing us from directly assessing potential hormonal modulation of glucose metabolism in AD. Future studies should investigate testosterone, estrogens, and gonadotropins as potential contributors to sex differences in AD neurobiology.

In conclusion, sex differences in regional brain glucose hypometabolism have been observed in AD, with women showing greater involvement in frontal and limbic regions and men in the associative parietal cortex. These differences suggest that biological and hormonal factors may influence the pathology and progression of the disease. Acknowledging these sex-specific variations could improve diagnostic accuracy and potentially inform the development of targeted therapies.

## Data Availability

The data generated and analyzed during the current study are available from the corresponding author to qualified investigators, upon reasonable request.

## References

[1] Alzheimer’s disease facts and figures. Alzheimers Dement. 2024;20:3708–821. 10.1002/alz.13809. 38689398 10.1002/alz.13809 PMC11095490

[2] Mielke MM, Vemuri P, Rocca WA. Clinical epidemiology of Alzheimer’s disease: assessing sex and gender differences. Clin Epidemiol. 2014;6:37–48. 10.2147/clep.S37929. 24470773 10.2147/CLEP.S37929 PMC3891487

[3] Snyder HM, et al. Sex biology contributions to vulnerability to Alzheimer’s disease: a think tank convened by the Women’s Alzheimer’s Research Initiative. Alzheimers Dement. 2016;12:1186–96. 10.1016/j.jalz.2016.08.004. 27692800 10.1016/j.jalz.2016.08.004 PMC10341380

[4] Ferretti MT, et al. Sex differences in Alzheimer disease - the gateway to precision medicine. Nat Rev Neurol. 2018;14:457–69. 10.1038/s41582-018-0032-9. 29985474 10.1038/s41582-018-0032-9

[5] Farrer LA, APOE and Alzheimer Disease Meta Analysis Consortium. Effects of age, sex, and ethnicity on the association between apolipoprotein E genotype and Alzheimer disease. A meta-analysis. JAMA. 1997;278:1349–56. 9343467

[6] Nebel RA, et al. Understanding the impact of sex and gender in Alzheimer’s disease: a call to action. Alzheimers Dement. 2018;14:1171–83. 10.1016/j.jalz.2018.04.008. 29907423 10.1016/j.jalz.2018.04.008 PMC6400070

[7] Altmann A, Tian L, Henderson VW, Greicius MD. Sex modifies the APOE-related risk of developing Alzheimer disease. Ann Neurol. 2014;75:563–73. 10.1002/ana.24135. 24623176 10.1002/ana.24135 PMC4117990

[8] Corder EH, et al. The biphasic relationship between regional brain senile plaque and neurofibrillary tangle distributions: modification by age, sex, and APOE polymorphism. Ann N Y Acad Sci. 2004;1019:24–8. 10.1196/annals.1297.005. 15246987 10.1196/annals.1297.005

[9] Livingston G, et al. Dementia prevention, intervention, and care: 2024 report of the Lancet standing Commission. Lancet. 2024;404:572–628. 10.1016/s0140-6736(24)01296-0. 39096926 10.1016/S0140-6736(24)01296-0

[10] Mosconi L, et al. Sex differences in Alzheimer risk: brain imaging of endocrine vs chronologic aging. Neurology. 2017;89:1382–90. 10.1212/wnl.0000000000004425. 28855400 10.1212/WNL.0000000000004425 PMC5652968

[11] McEwen B. Estrogen actions throughout the brain. Recent Prog Horm Res. 2002;57:357–84. 10.1210/rp.57.1.357. 12017552 10.1210/rp.57.1.357

[12] Rowe CC, et al. Amyloid imaging results from the Australian Imaging, Biomarkers and Lifestyle (AIBL) study of aging. Neurobiol Aging. 2010;31:1275–83. 10.1016/j.neurobiolaging.2010.04.007. 20472326 10.1016/j.neurobiolaging.2010.04.007

[13] Perneczky R, Drzezga A, Diehl-Schmid J, Li Y, Kurz A. Gender differences in brain reserve : an (18)F-FDG PET study in Alzheimer’s disease. J Neurol. 2007;254:1395–400. 10.1007/s00415-007-0558-z. 17934882 10.1007/s00415-007-0558-z

[14] Barnes LL, et al. Sex differences in the clinical manifestations of Alzheimer disease pathology. Arch Gen Psychiatry. 2005;62:685–91. 10.1001/archpsyc.62.6.685. 15939846 10.1001/archpsyc.62.6.685

[15] Nobili F, et al. European association of nuclear medicine and European academy of neurology recommendations for the use of brain (18) F-fluorodeoxyglucose positron emission tomography in neurodegenerative cognitive impairment and dementia: delphi consensus. Eur J Neurol. 2018;25:1201–17. 10.1111/ene.13728. 29932266 10.1111/ene.13728

[16] Shen X, Liu H, Hu Z, Hu H, Shi P. The relationship between cerebral glucose metabolism and age: report of a large brain PET data set. PLoS ONE. 2012;7:e51517. 10.1371/journal.pone.0051517. 23284706 10.1371/journal.pone.0051517 PMC3527454

[17] Fujimoto T, et al. Changes in glucose metabolism due to aging and gender-related differences in the healthy human brain. Psychiatry Res. 2008;164:58–72. 10.1016/j.pscychresns.2006.12.014. 18804967 10.1016/j.pscychresns.2006.12.014

[18] Willis MW, et al. Age, sex and laterality effects on cerebral glucose metabolism in healthy adults. Psychiatry Res. 2002;114:23–37. 10.1016/s0925-4927(01)00126-3. 11864807 10.1016/s0925-4927(01)00126-3

[19] Allocca M, et al. Evaluation of age and sex-related metabolic changes in healthy subjects: an Italian brain 18F-FDG PET study. J Clin Med. 2021. 10.3390/jcm10214932. 34768454 10.3390/jcm10214932 PMC8584846

[20] Kakimoto A, et al. Age-related sex-specific changes in brain metabolism and morphology. J Nucl Med. 2016;57:221–5. 10.2967/jnumed.115.166439. 26609179 10.2967/jnumed.115.166439

[21] Malpetti M, et al. Gender differences in healthy aging and Alzheimer’s dementia: a (18) F-FDG-PET study of brain and cognitive reserve. Hum Brain Mapp. 2017;38:4212–27. 10.1002/hbm.23659. 28561534 10.1002/hbm.23659 PMC6866811

[22] McKhann GM, et al. The diagnosis of dementia due to Alzheimer’s disease: recommendations from the National Institute on Aging-Alzheimer’s Association workgroups on diagnostic guidelines for Alzheimer’s disease. Alzheimers Dement. 2011;7:263–9. 10.1016/j.jalz.2011.03.005. 21514250 10.1016/j.jalz.2011.03.005 PMC3312024

[23] Giacomucci G, et al. Plasma p-tau181 as a promising non-invasive biomarker of Alzheimer’s disease pathology in subjective cognitive decline and mild cognitive impairment. J Neurol Sci. 2023;453:120805. 10.1016/j.jns.2023.120805. 37716237 10.1016/j.jns.2023.120805

[24] Mazzeo S, et al. Plasma neurofilament light chain predicts Alzheimer’s disease in patients with subjective cognitive decline and mild cognitive impairment: a cross-sectional and longitudinal study. Eur J Neurol. 2024;31:e16089. 10.1111/ene.16089. 37797300 10.1111/ene.16089 PMC11235835

[25] Nerattini M, et al. Cerebral amyloid load determination in a clinical setting: interpretation of amyloid biomarker discordances aided by tau and neurodegeneration measurements. Neurol Sci. 2022;43:2469–80. 10.1007/s10072-021-05704-2. 34739618 10.1007/s10072-021-05704-2

[26] Guedj E, et al. EANM procedure guidelines for brain PET imaging using [(18)F]FDG, version 3. Eur J Nucl Med Mol Imaging. 2022;49:632–51. 10.1007/s00259-021-05603-w. 34882261 10.1007/s00259-021-05603-w PMC8803744

[27] Friston KJ, et al. Statistical parametric maps in functional imaging: a general linear approach. Hum Brain Mapp. 1994;2:189–210. 10.1002/hbm.460020402.

[28] Della Rosa PA, et al. A standardized [18F]-FDG-PET template for spatial normalization in statistical parametric mapping of dementia. Neuroinformatics. 2014;12:575–93. 10.1007/s12021-014-9235-4. 24952892 10.1007/s12021-014-9235-4

[29] Yakushev I, et al. SPM-based count normalization provides excellent discrimination of mild Alzheimer’s disease and amnestic mild cognitive impairment from healthy aging. Neuroimage. 2009;44:43–50. 10.1016/j.neuroimage.2008.07.015. 18691659 10.1016/j.neuroimage.2008.07.015

[30] Caminiti SP, et al. Validation of FDG-PET datasets of normal controls for the extraction of SPM-based brain metabolism maps. Eur J Nucl Med Mol Imaging. 2021;48:2486–99. 10.1007/s00259-020-05175-1. 33423088 10.1007/s00259-020-05175-1

[31] Ashburner J, Friston KJ. Voxel-based morphometry–the methods. Neuroimage. 2000;11:805–21. 10.1006/nimg.2000.0582. 10860804 10.1006/nimg.2000.0582

[32] Rolls ET, Huang CC, Lin CP, Feng J, Joliot M. Automated anatomical labelling atlas 3. Neuroimage. 2020;206:116189. 10.1016/j.neuroimage.2019.116189. 31521825 10.1016/j.neuroimage.2019.116189

[33] Bergamino M, et al. Sex differences in Alzheimer’s disease revealed by free-water diffusion tensor imaging and voxel-based morphometry. J Alzheimers Dis. 2022;85:395–414. 10.3233/jad-210406. 34842185 10.3233/JAD-210406 PMC9015709

[34] Hanyu H, et al. Differences in regional cerebral blood flow patterns in male versus female patients with Alzheimer disease. AJNR Am J Neuroradiol. 2004;25:1199–204. 15313710 PMC7976554

[35] Braak H, Braak E. Neuropathological stageing of Alzheimer-related changes. Acta Neuropathol. 1991;82:239–59. 10.1007/bf00308809. 1759558 10.1007/BF00308809

[36] Edwards L, et al. Multimodal neuroimaging of sex differences in cognitively impaired patients on the Alzheimer’s continuum: greater tau-PET retention in females. Neurobiol Aging. 2021;105:86–98. 10.1016/j.neurobiolaging.2021.04.003. 34049062 10.1016/j.neurobiolaging.2021.04.003 PMC8820163

[37] Morrison JH, Brinton RD, Schmidt PJ, Gore AC. Estrogen, menopause, and the aging brain: how basic neuroscience can inform hormone therapy in women. J Neurosci. 2006;26:10332–48. 10.1523/jneurosci.3369-06.2006. 17035515 10.1523/JNEUROSCI.3369-06.2006 PMC6674699

[38] Siddiqui AN, et al. Neuroprotective role of steroidal sex hormones: an overview. CNS Neurosci Ther. 2016;22:342–50. 10.1111/cns.12538. 27012165 10.1111/cns.12538 PMC6492877

[39] Ciocca G, et al. Is testosterone a food for the brain? Sex Med Rev. 2016;4:15–25. 10.1016/j.sxmr.2015.10.007. 27872000 10.1016/j.sxmr.2015.10.007

[40] Nerattini M, et al. Sex-specific associations of serum testosterone with gray matter volume and cerebral blood flow in midlife individuals at risk for Alzheimer’s disease. PLoS ONE. 2025;20:e0317303. 10.1371/journal.pone.0317303. 39804890 10.1371/journal.pone.0317303 PMC11729972

[41] Ford AH, et al. Sex hormones and incident dementia in older men: the health in men study. Psychoneuroendocrinology. 2018;98:139–47. 10.1016/j.psyneuen.2018.08.013. 30144781 10.1016/j.psyneuen.2018.08.013

[42] Marriott RJ, et al. Lower serum testosterone concentrations are associated with a higher incidence of dementia in men: The UK Biobank prospective cohort study. Alzheimers Dement. 2022;18:1907–18. 10.1002/alz.12529. 34978125 10.1002/alz.12529

[43] Paganini-Hill A, Henderson VW. Estrogen deficiency and risk of Alzheimer’s disease in women. Am J Epidemiol. 1994;140:256–61. 10.1093/oxfordjournals.aje.a117244. 8030628 10.1093/oxfordjournals.aje.a117244

[44] Manly JJ, et al. Endogenous estrogen levels and Alzheimer’s disease among postmenopausal women. Neurology. 2000;54:833–7. 10.1212/wnl.54.4.833. 10690972 10.1212/wnl.54.4.833

[45] Bowen RL, Isley JP, Atkinson RL. An association of elevated serum gonadotropin concentrations and Alzheimer disease? J Neuroendocrinol. 2000;12:351–4. 10.1046/j.1365-2826.2000.00461.x. 10718932 10.1046/j.1365-2826.2000.00461.x

[46] Casadesus G, et al. Beyond estrogen: targeting gonadotropin hormones in the treatment of Alzheimer’s disease. Curr Drug Targets CNS Neurol Disord. 2004;3:281–5. 10.2174/1568007043337265. 15379604 10.2174/1568007043337265

[47] Xiong J, et al. FSH blockade improves cognition in mice with Alzheimer’s disease. Nature. 2022;603:470–6. 10.1038/s41586-022-04463-0. 35236988 10.1038/s41586-022-04463-0 PMC9940301

[48] Nerattini M, et al. Elevated gonadotropin levels are associated with increased biomarker risk of Alzheimer’s disease in midlife women. Frontiers in Dementia. 2023. 10.3389/frdem.2023.1303256. 38774256 10.3389/frdem.2023.1303256 PMC11108587

[49] Maioli S, Leander K, Nilsson P, Nalvarte I. Estrogen receptors and the aging brain. Essays Biochem. 2021;65:913–25. 10.1042/ebc20200162. 34623401 10.1042/EBC20200162 PMC8628183

[50] Mosconi L, et al. Reduced hippocampal metabolism in MCI and AD: automated FDG-PET image analysis. Neurology. 2005;64:1860–7. 10.1212/01.Wnl.0000163856.13524.08. 15955934 10.1212/01.WNL.0000163856.13524.08

[51] Hamilton, C. *Cognition and sex differences*. (Bloomsbury Publishing, 2008)

[52] Christov-Moore L, et al. Empathy: gender effects in brain and behavior. Neurosci Biobehav Rev. 2014;46(Pt 4):604–27. 10.1016/j.neubiorev.2014.09.001. 25236781 10.1016/j.neubiorev.2014.09.001 PMC5110041

[53] Mosconi L, et al. Menopause impacts human brain structure, connectivity, energy metabolism, and amyloid-beta deposition. Sci Rep. 2021;11:10867. 10.1038/s41598-021-90084-y. 34108509 10.1038/s41598-021-90084-y PMC8190071

[54] Mosconi L, et al. In vivo brain estrogen receptor density by neuroendocrine aging and relationships with cognition and symptomatology. Sci Rep. 2024;14:12680. 10.1038/s41598-024-62820-7. 38902275 10.1038/s41598-024-62820-7 PMC11190148

[55] Lyketsos CG, Olin J. Depression in Alzheimer’s disease: overview and treatment. Biol Psychiatry. 2002;52:243–52. 10.1016/s0006-3223(02)01348-3. 12182930 10.1016/s0006-3223(02)01348-3

